# Unravelling the relevance of CLEC12A as a cancer stem cell marker in myelodysplastic syndrome

**DOI:** 10.1111/bjh.14270

**Published:** 2016-09-09

**Authors:** Marie Toft‐Petersen, Line Nederby, Eigil Kjeldsen, Gitte B. Kerndrup, Gordon D. Brown, Peter Hokland, Anne Stidsholt Roug

**Affiliations:** ^1^ Department of HaematologyAarhus University HospitalAarhusDenmark; ^2^ Department of PathologyAarhus University HospitalAarhusDenmark; ^3^ ImmunityInfection and Inflammation ProgrammeInstitute of Medical SciencesUniversity of AberdeenAberdeenUK

**Keywords:** cancer stem cells, myelodysplastic syndrome, hMICL, CD371, LTC‐IC

## Abstract

Evidence of distinct disease propagating stem cells in myelodysplastic syndrome (MDS) has emerged in recent years. However, immunophenotypic characterization of these cancer stem cells remains sparse. In acute myeloid leukaemia (AML), we have previously described aberrant expression of the C‐type lectin domain family 12, member A (CLEC12A) as a stable and reliable marker of leukaemia blasts and as a tool for assessing minimal residual disease. Furthermore, CLEC12A has been proposed as a promising marker of leukaemic stem cells in AML. The role of CLEC12A in MDS, however, remains to be elucidated. In this study, we found CLEC12A aberrantly expressed on the CD34^+^ CD38^−^ cell compartment in 71% (22/31) of MDS patients, distributed across all Revised International Prognostic Scoring System risk groups. We showed that the CD34^+^ CD38^−^ CLEC12A^+^ cells were indeed malignant and possessed functional stem cell properties in the long‐term colony‐initiating cell assay. As opposed to reported findings in AML, we showed that cancer stem cells from MDS samples derived from both CLEC12A positive and negative CD34^+^ CD38^−^ subpopulations. Due to the absence of CLEC12A on normal haematopoietic stem cells, CLEC12A stem cell immunophenotyping may contribute to diagnosing and monitoring MDS patients and could furthermore add knowledge about disease propagating cells in MDS.

Myelodysplastic syndrome (MDS) is a heterogeneous group of clonal diseases marked by dysplasia in the bone marrow (BM) causing cytopenias in the peripheral blood (PB) and with an inherent tendency to progress to acute myeloid leukaemia (AML). Diagnosis remains a challenge and relies on morphological and cytogenetic evaluation after the exclusion of other causes of cytopenias. When the morphological dysplastic features are mild or even absent and PB cytopenias are persistent the diagnosis is termed idiopathic cytopenias of undetermined significance (ICUS) (Valent *et al*, 2012; Bejar & Steensma, 2014). Evaluating myelodysplastic features using flow cytometry is a growing field of interest and highly sensitive multicolour immunophenotypic profiling and accompanying scoring systems have proven useful in MDS, both in diagnostic and prognostic terms (Ogata *et al*, 2006; van de Loosdrecht *et al*, 2008).

The concept of the leukaemic stem cell (LSC) is well established in AML and the frequency of the CD34^+^CD38^−^ stem cell compartment with aberrant marker expression has proven predictive of adverse outcome in several studies (Vergez *et al*, 2011; Terwijn *et al*, 2014). Following the seminal studies by Bakker *et al* (2004), we have identified the C‐type lectin domain family 12, member A (CLEC12A) (also named hMICL and CLL‐1) as a stable and reliable leukaemia‐associated marker at diagnosis and as a tool for assessing minimal residual disease in AML (Larsen *et al*, 2012; Roug *et al*, 2014). Furthermore, CLEC12A has been proposed as a possible marker for LSC as it can be found on CD34^+^CD38^−^ cells in CD34‐positive AML, but is completely absent on the CD34^+^CD38^−^ compartment in normal donors and in regenerating BM (van Rhenen *et al*, 2007a). In addition, Terwijn *et al* (2014) showed that light scatter properties together with aberrant marker expression (including CLEC12A) on the CD34^+^CD38^−^ cells were able to discriminate leukaemic and normal stem cell compartments in AML validated by xenograft studies.

Regarding the clonal origin of MDS, the disease entity has generally been regarded as originating in the haematopoietic stem cell (HSC), which recent research has emphasized to hold true also for low‐risk MDS (Tehranchi *et al*, 2010). Furthermore, for the 5q‐ syndrome, it has been shown that, even at an early time point in the course of the disease, the normal HSC compartment has almost completely been replaced with clonally expanded stem cells that are positive for the cytogenetic 5q‐ aberration (Nilsson *et al*, 2007). Woll *et al* (2014) provided evidence of distinct cancer stem cells (CSCs) in MDS by tracing all identified somatic mutations in the bulk BM back to the immunophenotypically defined Lin‐CD34^+^CD38^−^CD90^+^ stem cell. Furthermore, this particular stem cell subset was the only cell type with functional self‐renewal potential in the long‐term colony initiating cell (LTC‐IC) assay and hence the ability to maintain and propagate the myelodysplastic neoplasm (Woll *et al*, 2014). While the above mentioned studies investigated stem cell subsets defined by the normal immunophenotypic profile of the HSC, other research groups have shown aberrant marker‐expression on CD34^+^CD38^−^ cells in MDS, although only in minor fractions of patients (Florian *et al*, 2006; Xie *et al*, 2010; Li *et al*, 2014).

Given the formal demonstration of CSCs in MDS (Woll *et al*, 2014), and the promising findings of CLEC12A as a surrogate marker of LSCs in AML (van Rhenen *et al*, 2007a), we hypothesized CLEC12A could be used as an immunophenotypic marker of CSCs in MDS. Consequently, we examined the aberrant expression of CLEC12A in the CD34^+^CD38^−^ cell compartment in BM from a cohort of MDS patients. Furthermore, to functionally validate the stem cell potential and malignant nature of these particular cells, we employed a two‐pronged strategy by; i) evaluating the self‐renewal properties of both the CD34^+^CD38^−^CLEC12A^+/−^ subsets in the LTC‐IC assay and ii) performing interphase fluorescence in situ hybridization (FISH) analyses of the derived colony‐forming cells (CFCs).

## Methods

### Patient samples

Bone marrow samples were obtained as excess material taken as part of the diagnostic process from 31 MDS patients diagnosed at the Department of Haematology, Aarhus University Hospital. The study was conducted in accordance with the Declaration of Helsinki and was approved by the local ethics committee. All samples were collected before treatment with cytoreductive or demethylating agents. MDS diagnoses were confirmed by morphological examination according to the 2008 World Health Organization (WHO) classification (Swerdlow *et al*, 2008). Risk assessment was done according to the Revised International Prognostic Scoring System (IPPS‐R) (Greenberg *et al*, 2012). In the immunophenotypic assay, normal BM (NBM) from 19 healthy volunteers and BM from 27 AML patients served as negative and positive controls, respectively.

### Flow cytometry

Bone marrow was analysed either fresh (MDS patients 1–19, NBM 1–11, AML patients 1–8) after red blood cells were lysed using EasyLyse (DAKO, Glostrup, Denmark) or as thawed cryopreserved mononuclear cells (MNCs) (MDS patients 20–31, NBM 12–19, AML patients 9–27). MNCs were obtained by Lymphoprep (Axis‐Shield plc., Dundee, Scotland) separation according to the manufacturer's instructions, cryopreserved in 10% dimethylsulfoxide and stored in liquid nitrogen. Cryopreserved MNCs were thawed in a 37°C water bath and resuspended in RoboSep Buffer (StemCell Technologies, Vancouver, BC, Canada) with 15% heat inactivated fetal calf serum (Biochrom, GmbH, Berlin, Germany). Subsequently, cells were stained with the following monoclonal antibodies: anti‐CD34 allophycocyanin (Birma‐K3), anti‐CD38 fluorescein‐isothiocyanate (AT 13/5) (DAKO), anti‐CD45 peridinin‐chlorophyll‐protein‐cyanine5·5 (HI30) (BioLegend, San Diego, CA, USA) and anti‐CLEC12A phycoerythrin (HB3) (own laboratory, courtesy of Gordon D. Brown). The anti‐CLEC12A antibody specificity was previously validated by Marshall *et al* (2006) and Larsen *et al* (2012). Data acquisition was performed on a FACSCanto II (BD Biosciences, San Jose, CA, USA) and analysed using FlowJo Data Analysis Software, version X (FlowJo, Ashland, OR, USA). In the gating strategy used, the CD34^+^CD38^−^ population was identified in CD45lowSSClow leucocytes and analysed for the presence/absence of CLEC12A. The CLEC12A positive gate was defined using the lymphocytes as an internal negative control. The CD34^+^CD38^−^CLEC12A^+^ population was back‐gated and was shown to cluster together in a forward scatter – side scatter plot. A minimum of five clustered CD34^+^CD38^−^CLEC12A^+^ events was considered significant.

### Fluorescence activated cell sorting

In four selected MDS patient cases (MDS patients 8, 29, 30 and 31) fluorescence activated cell sorting (FACS) was performed on a BD FACSAria^™^ III (BD Biosciences). Cells were sorted into two subsets, CD45lowSSClowCD34^+^CD38^−^CLEC12A^+^ and CD45lowSSClowCD34^+^CD38^−^CLEC12A^−^ using the same gating strategy as described above. The CD45lowSSClowCD34^+^CD38^−^CLEC12A^−^ subset from two NBMs was sorted as controls. Purity of sorted subsets was determined in 5 out of 10 subsets due to the rarity of the cell populations. For the CD45lowSSClowCD34^+^CD38^−^CLEC12A^−^ subset, purity was >99% (*n* = 4; MDS patient 8, 30, and 31, NBM 1). For the CD45lowSSClowCD34^+^CD38^−^CLEC12A^+^ subset, purity was 91·3% (*n* = 1; MDS patient 30).

### Long‐term colony initiating cell assay

Fluorescence activated cell sorted subsets were seeded in up to four replicates in 96‐well collagen‐coated plates and cultured on irradiated (80 Gy) M2‐10B4 stromal feeder cells (American Type Culture Collection [ATCC], Manassas, VA, USA) in MyeloCult H5100 medium (StemCell Technologies, Vancouver, BC, Canada) supplemented with penicillin‐streptomycin (Gibco^®^, Thermo Fischer Scientific Inc., Waltham, MA, USA) and 10^−6^ mol/l hydrocortisone 21‐hemisuccinate (StemCell Technologies) with weekly half‐change of medium. After 6 weeks of co‐culture, cells were harvested and transferred to MethoCult H4435 (StemCell Technologies) for an additional 2 weeks. CFCs were counted and scored for multi‐lineage (colony‐forming unit‐granulocyte/erythroid/macrophage/megakaryocyte (CFU‐GEMM)); erythroid (burst‐forming unit ‐ erythrocyte (BFU‐E)) and myeloid morphology [colony‐forming unit ‐ granulocyte (CFU‐G), colony‐forming unit ‐ macrophage (CFU‐M) and colony‐forming unit ‐ granulocyte/macrophage (CFU‐GM)]. Ten colonies from each subset of the originally seeded cells were individually picked and cytospun onto poly‐L‐lysine coated slides (Thermo Fischer Scientific Inc., Waltham, MA, USA) and allowed to air‐dry for subsequent FISH analyses.

### Interphase fluorescence in situ hybridization

FISH analyses were done according to manufacturer's instructions except that all slides containing the cells were pre‐treated with pepsin for 3·5 min (Histology FISH accessory kit, DAKO, Glostrup, Denmark). The following locus‐specific directly fluorescent‐labelled probe set were used: 7pter; D8Z1 (Kreatech, Amsterdam, The Netherlands); MYC split apart probe (DAKO); D1Z5; LSI EGR1/D5S23,D5S721 and LSI ELN/D7S486,D7S522 (Abbott Molecular, Weissbaden, Germany). To estimate the number of FISH‐positive cells, a median of 100 interphase nuclei were evaluated (range 3–100) by two independent observers. Imaging was done as described by Kjeldsen and Roug (2012). Cytospin slides of CFCs from NBM served as negative controls for each of the specific probes used.

### Statistical analyses

All calculations and statistical analyses were conducted with the use of GraphPad Prism version 6 (GraphPad Software, La Jolla, CA, USA). The Mann‐Whitney test was used to compare the level of CD34^+^CD38^−^CLEC12A^+^ in untreated MDS BM to NMB and AML BM. Kaplan‐Meier estimates, together with the log‐rank test were used to study the impact of a detectable fraction of CLEC12A+ cells in the CD34^+^CD38^−^ cell compartment on overall survival (OS) and AML‐free survival. OS was calculated from the sampling date to the date of death by any cause. AML‐free survival was calculated from the sampling date to the date of progression to AML or death by any cause. *P* < 0·05 was considered significant.

## Results

### Demographic data

The median age of the MDS patients was 69 years (range 29–83 years) at the time of sampling. The MDS subtype according to the 2008 WHO Classification, karyotype, IPSS‐R risk group as well as AML‐free survival are depicted in Table 1. As evident herein, all subtypes of MDS were represented and the patients were distributed across all IPSS‐R risk groups. Seventeen patients (55%) presented with a normal karyotype, which is in accordance with the literature (Bejar & Steensma, 2014).

**Table 1:** Patient characteristics

Patient no.	WHO subtype	Clonal chromosomal abnormalities detected	IPSS‐R Risk group	CD34^+^CD38^−^CLEC12A^+^/ CD34^+^CD38^−^	AML‐free survival (d)
1	RAEB‐1	No aberrations	LOW	0%	
2	RAEB‐1	No aberrations	INT	0%	605
3	RCMD	No aberrations	V‐LOW	0%	
4	RCUD	+8	LOW	37·9%	1169
5	RAEB‐1	inv(3)(p12q26)	V‐HIGH	6·3%	583
6	RAEB‐1	No aberrations	INT	56·7%	16
7	RCUD	Complex	INT	0%	
8	RAEB‐1	+del(1)(p13), +8	INT	1·5%	658
9	RCUD	No aberrations	V‐LOW	17·4%	154
10	NA^a^	ND	NA	1·8%	713
11	Hypo RCUD	No aberrations	LOW	4·1%	91
12	RAEB‐1	No aberrations	HIGH	0%	
13	RAEB‐1	No aberrations	INT	6·4%	
14	RCUD	‐X	INT	22·6%	
15	RCUD	No aberrations	LOW	11·6%	
16	RCUD	No aberrations	V‐LOW	14·7%	
17	RARS	No aberrations	LOW	0%	
18	MDS‐U	No aberrations	LOW	3·2%	210
19	RAEB‐2	No aberrations	HIGH	4·3%	
20	RCUD	del(15)(q22q26)	HIGH	5·6%	
21	RAEB‐2	Complex, incl. del(5q)(q13q33),‐7	V‐HIGH	0·6%	55
22	RCUD	‐7	INT	5·6%	
23	RCUD	No aberrations	LOW	1·2%	
24	RCUD	No aberrations	V‐LOW	6·8%	405
25	MDS‐U	+mar	LOW	0%	670
26	RCUD	No aberrations	INT	0%	554
27	RCUD	‐Y	V‐LOW	0%	
28	RCUD	No aberrations	V‐LOW	7·4%	
29	RCUD	+8	INT	12·0%	482
30	RCMD	Complex, incl. ‐5, ‐7	NA^a^	17·4%	
31	Del(5q)	del(5)(q13q31)	INT	0·1%	

AML, acute myeloid leukaemia; INT, intermediate; IPSS‐R, Revised International Prognostic Scoring System; MDS‐U, myelodysplastic syndrome – unclassified; NA, not applicable; ND, not done; RAEB‐1, refractory anaemia with excess blasts type 1; RAEB‐2, refractory anaemia with excess blasts type 2; RARS, refractory anaemia with ringed sideroblasts; RCMD, refractory cytopenia with multilineage dysplasia; RCUD, refractory cytopenia with unilineage dysplasia; V‐HIGH, Very High; V‐LOW, Very Low; WHO, World Health Organization.

a Insufficient material for complete morphological evaluation.

### CLEC12A is aberrantly expressed on the MDS stem cell subset

**Figure 1:**
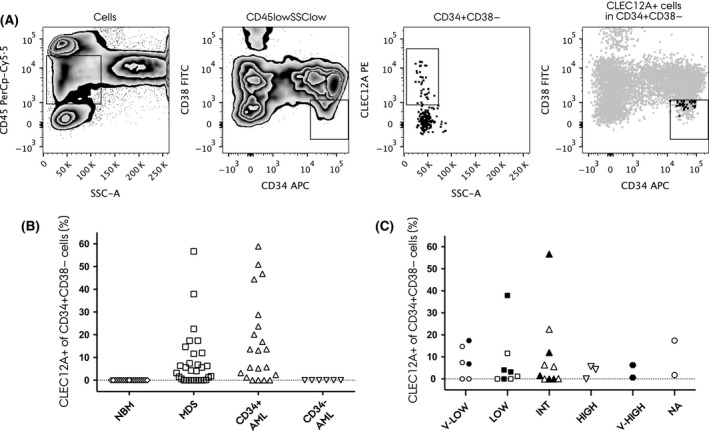
CLEC12A expression on the CD34^+^ CD38^−^ stem cell subset. (A) Gating strategy (MDS Patient 9). In the first panel, the CD45low/SSClow population was defined. Next, the CD34^+^ CD38^−^ population was gated and analysed for the presence/absence of CLEC12A. The last panel shows the CD34^+^ CD38^−^ CLEC12A^+^ population back‐gated into the CD34^+^ CD38^−^ plot. (B) CLEC12A expression on the CD34^+^ CD38^−^ cell subset in normal bone marrow (NBM), myelodysplastic syndrome (MDS), CD34‐positive (CD34^+^) acute myeloid leukaemia (AML) and CD34‐negative (CD34^−^) AML, respectively. (C) CLEC12A expression on the CD34^+^ CD38^−^ cell subset according to IPSS‐R risk groups. Filled symbols indicate later progression to AML.

We initially set out to determine the immunophenotypic profile of the stem cell subset CD45lowCD34^+^CD38^−^ with regards to co‐expression of CLEC12A. The gating strategy for the assay is depicted in Fig 1A. Consistent with previous findings (van Rhenen *et al*, 2007a), CLEC12A was completely absent on the CD34^+^CD38^−^ cell compartment in all of the 19 NBM examined (0·0%). In contrast, we found CLEC12A to be aberrantly expressed on the CD34^+^CD38^−^ cell subset in 71% (22/31) of MDS cases. In myeloid neoplasms, the presence of several malignant subclones and evidence of clonal evolution during the course of disease have been studied by several groups (Fialkow *et al*, 1991; Walter *et al*, 2012). Thus, we found it appealing to investigate the aberrant CD34^+^CD38^−^CLEC12A^+^ subset as a fraction of the immature CD34^+^CD38^−^ cells. Applying this method, the median percentage of aberrant cells as a fraction of the CD34^+^CD38^−^ subset was 4·1% (range 0·0–56·7%), which was significantly different from NBM (*P* < 0·0001).

Similarly, for the AML control samples, we determined the fractions of the CLEC12A^+^ subsets as a percentage of the CD34^+^CD38^−^ subset. In CD34‐positive AML (*n* = 21) (defined as >2% CD34^+^ cells of the blast population), the percentage of CLEC12A‐positive CD34^+^CD38^−^ cells amounted a median of 13·1% (range 0·0–58·9%), which was significantly different from NBM (*P* < 0·0001), but not from MDS BM (*P* = 0·08) (Fig 1B). In BM samples with a detectable CLEC12A^+^ fraction of the CD34^+^CD38^−^ cells, there was no significant difference in median fluorescence intensity of CLEC12A between CD34‐positive AML and MDS (*P* = 0·12). As expected, expression of CLEC12A was not found on the CD34^+^CD38^−^ cells in CD34‐negative AML (*n* = 6) (Fig 1B). Hence, although with a p‐value approaching significance in favour of a higher amount of CLEC12A expressing CD34^+^CD38^−^ cells in CD34^+^ AML, in our cohort the level of this particular cell subset is seemingly comparable in MDS and CD34^+^ AML. When we evaluated the expression of CLEC12A on the CD34^+^CD38^−^ compartment across IPPS‐R risk groups, we found it to be present in all of these and thus not restricted to cases with excess blasts or high‐risk MDS (Fig 1C).

Collectively, the immunophenotypic data support the hypothesis of CLEC12A as an aberrantly expressed marker on the stem cell subset in MDS. Furthermore, this finding was not restricted to high risk MDS or MDS with excess blasts, as could be surmised from the established fact of CLEC12A as a marker of blasts and LSCs in AML, suggesting a role for this marker in the broad spectrum of myelodysplastic disorders.

### The CD34^+^CD38^−^CLEC12A^+^ cells are malignant and possess self‐renewal potential

While the above‐described data clearly infers that CLEC12A can be expressed on stem cells in MDS, this does not constitute definite evidence of the functional stem cell properties or the malignancy of these particular cells. Primary human MDS cells grow poorly in most mouse models and highly specific and genetically engineered mouse models are required to study human MDS in the xenograft setting (Zhou *et al*, 2015). Consequently, to functionally assess the self‐renewal potential of both the CLEC12A positive CD34^+^CD38^−^ cells and the CD34^+^CD38^−^ cells lacking CLEC12A expression, four MDS patients with known clonal cytogenetic abnormalities were selected for studies in the LTC‐IC assay. The cytogenetic abnormalities of the patients are depicted in Table 2 together with the number of seeded cells from each sorted cell subset, CFC counts and origin (erythroid/myeloid), and FISH results.

**Table 2:** Results from LTC‐IC and subsequent FISH analyses

Patient	Targeted chromosomal aberrations (FISH probe locus)	Sorted subsets	Seeded in LTC‐IC	CFC count^a^		FISH positivity
				Erythroid	Myeloid	
MDS 8	+1q (CEP1) and +8 (SE8)	CD34^+^CD38^−^CLEC12A^+^	19	0	24	10/10 (100%)
		CD34^+^CD38^−^CLEC12A^−^	500	1	410	10/10 (100%)
MDS 29	+8 (MYC)	CD34^+^CD38^−^CLEC12A^+^	146	0	40	7/7 (100%)
		CD34^+^CD38^−^CLEC12A^−^	500	2	34	2/10 (20%)^b^
MDS 30	‐5 (EGR1) and ‐7 (D7S486,D7S522)	CD34^+^CD38^−^CLEC12A^+^	500	0	245	10/10 (100%)
		CD34^+^CD38^−^CLEC12A^−^	500	0	438	10/10 (100%)
MDS 31	‐5 (EGR1)	CD34^+^CD38^−^CLEC12A^+^	123	0	1^c^	No cells
		CD34^+^CD38^−^CLEC12A^−^	500	0	63	7/7 (100%)
NBM 1	(CEP1), (SE8)	CD34^+^CD38^−^CLEC12A^−^	500	2	19	No aberrations^e^
NBM 2	(EGR1), (D7S486,D7S522)	CD34^+^CD38^−^CLEC12A^−^	500	4	28^d^	No aberrations^e^

CFC, colony‐forming cell; CFU‐GEMM, colony‐forming unit‐granulocyte/erythroid/macrophage/megakaryocyte; FISH, fluorescence in situ hybridization; LTC‐IC, long‐term colony initiating cell assay; MDS, myelodysplastic syndrome; NBM, normal bone marrow.

a Mean CFC count of 2–3 plates normalized to 500 cells, when ≥500 cells originally seeded in LTC‐IC.

b FISH‐negative CFCs were morphologically normal.

c Scattered cells.

d In addition 1 CFU‐GEMM.

e NBM served as negative controls for all of the applied FISH‐probes.

**Figure 2:**
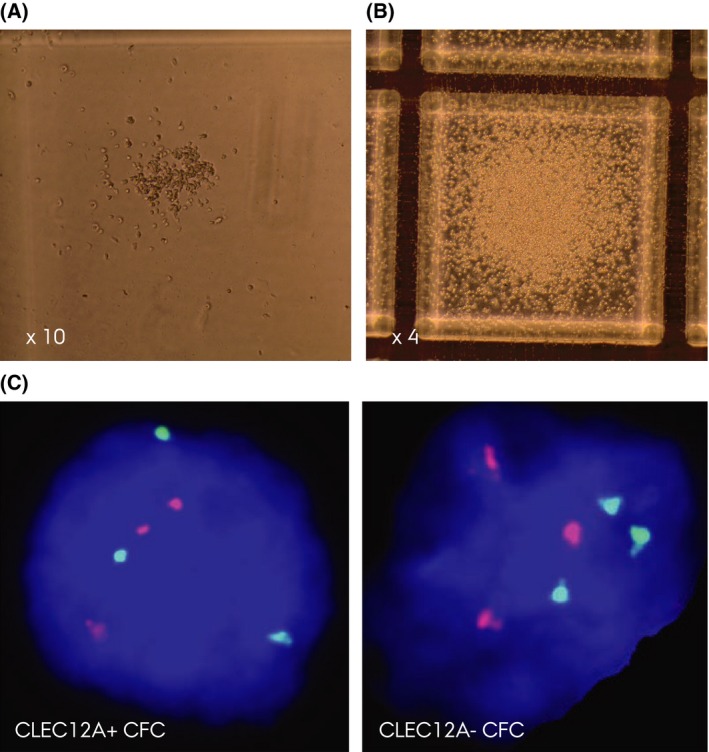
LTC‐IC and confirmatory FISH. (A) Representative colony‐forming cells (CFC) from the CD34^+^ CD38^−^ CLEC12A^+^ subset in MDS Patient 30. The colony is smaller and contains fewer cells than myeloid colonies from normal bone marrow (NBM). (B) Representative CFC from NBM. (C) Confirmatory fluorescence in situ hybridization using the D1Z5 probe (red) and centromeric D8Z1 probe (green) confirming the trisomy 1 and 8 aberrations (MDS Patient 8) in interphase nuclei (3R3G signal pattern) in a cell from the CD34^+^ CD38^−^ CLEC12A^+^ and CD34^+^ CD38^−^ CLEC12A^−^ derived colonies, respectively.

When we examined and harvested the single colonies derived from LTC‐IC, we observed that CFCs from MDS patients (both CLEC12A^+^ and CLEC12A^−^ subsets) were morphologically abnormal and smaller than those from normal controls and were almost exclusively of non‐erythroid origin (Fig 2A and B). Interestingly, erythroid CFCs were never present in the CD34^+^CD38^−^CLEC12A^+^ subset in any of the 4 MDS patients studied in LTC‐IC. This is in line with the general perception of CLEC12A as a myeloid marker.

In all harvested colonies analysed by FISH, 100% of CFCs from the CD34^+^CD38^−^CLEC12A^+^ subsets were of malignant origin as evidenced by the presence of specific cytogenetic aberrancies (Table 2). Interestingly, for MDS Patient 29, CFCs from the CD34^+^CD38^−^CLEC12A^−^ subset were a mixture of the aforementioned abnormal small colonies and morphologically normal CFCs. This observation was supported by and in accordance with the FISH results because the small abnormal CFCs were positive for trisomy 8 and the morphologically normal CFCs presented with a normal karyotype (Table 2). In the remaining samples examined in LTC‐IC, CFCs originating from the CD34^+^CD38^−^CLEC12A^−^ subsets were also 100% malignant when cytogenetically evaluated by FISH (Fig 2C). Overall, the data from the LTC‐IC studies showed the CD34^+^CD38^−^CLEC12A^+^ to possess self renewal properties and that these particular cells are 100% malignant when evaluated by FISH for known cytogenetic aberrancies, adding support to the concept of CLEC12A as a marker of malignant CSC subsets in MDS.

### The presence of CD34^+^CD38^−^CLEC12A^+^ cells and survival

Given that CLEC12A was expressed on CD34^+^CD38^−^ cells across all IPSS‐R risk groups, we looked at the impact of these cell subsets on the clinical course of the patients studied. The median follow up time was 659 d (range 56–2926 d). Progression to AML occurred in 45% (14/31) of cases, and 79% (11/14) of these had a detectable CD34^+^CD38^−^CLEC12A^+^ fraction at the time of sampling (Table 1). Survival analysis showed no correlation between the detection of CLEC12A expression on CD34^+^CD38^−^ cells and neither OS (*P* = 0·50) nor AML‐free survival (*P* = 0·12).

## Discussion

The present study showed that, as in AML, CLEC12A is present on the CD34^+^CD38^−^ cell compartment in the majority of the 31 MDS cases studied. Moreover, CLEC12A could be found expressed on a fraction of the CD34^+^CD38^−^ cells in all WHO subtypes of MDS and across the IPPS‐R risk groups. The percentage of CLEC12A^+^ cells in the CD34^+^CD38^−^ compartment in MDS was comparable to the level found in CD34‐positive AML. Conversely, expression of CLEC12A was not found on the CD34^+^CD38^−^ cells in CD34‐negative AML, which is consistent with previous findings indicating that in CD34‐negative AML, the small fraction of CD34‐positive cells in most cases represents normal HSCs (van der Pol *et al*, 2003; Taussig *et al*, 2010). Furthermore, and importantly, we showed that the CD34^+^CD38^−^CLEC12A^+^ cells are indeed malignant and possess functional stem cell properties in terms of self‐renewal when evaluated in the LTC‐IC assay.

In AML, evidence of the ability of CLEC12A to distinguish between the LSC and its normal counterpart, the HSC, was, to a certain extent, raised by van Rhenen *et al* (2007a,b), who showed that sorted CD34^+^CD38^−^CLEC12A^+^ cells were malignant by FISH, while the CD34^+^CD38^−^CLEC12A^−^ cells proved negative for the AML‐specific cytogenetic abnormalities. Furthermore, malignant engraftment of CD34^+^CLEC12A^+^ cells in non‐obese diabetic/severe combined immunodeficiency (NOD/SCID) mice was demonstrated. The immunophenotypic studies included patients with refractory anaemia with excess blasts (RAEB) and RAEB in transformation [defined by the French‐American‐British (FAB) classification (Bennett *et al*, 1982)], while the FISH analyses and the engraftment studies were done only on AML samples (van Rhenen *et al*, 2007a,b). Thus, to the best of our knowledge, functional studies of CLEC12A as a marker of CSCs in MDS have not been performed up until now. In the present study, we have corroborated the immunophenotypic findings of van Rhenen *et al* (2007a,b) by confirming the possible aberrant expression of CLEC12A on the CD34^+^CD38^−^ stem cell subset from MDS patients. In addition, we have extended these findings; firstly by demonstrating aberrant CD34^+^CD38^−^CLEC12A^+^ cells in all MDS subtypes defined by the current 2008 WHO classification and across all IPSS‐R risk groups, hence providing evidence that this phenomenon is not restricted to high‐risk MDS or MDS with excess blasts. This suggests CLEC12A to have an independent role as a CSC marker in MDS and not solely associated with AML, high‐risk MDS or MDS in transformation to AML. Secondly, by performing functional LTC‐IC studies, we observed notable differences between MDS and the previous findings in AML. As colonies derived from CD34^+^CD38^−^CLEC12A^−^ cells were predominantly malignant, CLEC12A was not able to distinguish between normal HSCs and CSCs, which might reflect important differences in disease biology between the two disease entities. Whereas MDS has been shown to originate from the earliest haematopoietic stem cell (Lin‐CD34^+^CD38^−^CD90^+^) (Woll *et al*, 2014), recent evidence suggest the majority of AML cases to propagate from more mature progenitors (Mora‐Jensen *et al*, 2015). Furthermore, in AML more than one LSC population can co‐exist in the same patient and be hierarchically ordered (Goardon *et al*, 2011). Speculatively, in MDS, CD34^+^CD38^−^CLEC12A^+^ cells could be a distinct CSC population downstream in a CSC hierarchy.

Our results from the LTC‐IC experiments therefore support the prevailing concept of MDS originating in the CD34^+^CD38^−^ subset. Interestingly, in the case of MDS Patient 29 only 20% of the CD34^+^CD38^−^CLEC12A^−^ CFCs were trisomy 8 positive, indicating a relatively large proportion of remaining normal HSCs in the BM of this particular patient. In line with this finding, Woll *et al* (2014) showed the frequency of Lin‐CD34^+^CD38^−^CD90^+^ cells harbouring the trisomy 8 aberration to be < 80%, while for the 5q‐ cases the stem cell compartment was almost entirely replaced by the malignant clone. Thus, our results also substantiate that residual normal HSCs ‐ if present ‐ are always contained in the CD34^+^CD38^−^CLEC12A^−^ cell subset.

Importantly, given the complete absence of CLEC12A on normal CD34^+^CD38^−^ cells, evaluating CLEC12A expression on CD34^+^CD38^−^ cells might be a useful diagnostic tool in MDS, representing a “different from normal”‐approach. Diagnosis of MDS stays a challenge and our study shows that CD34^+^CD38^−^CLEC12A^+^ cells are also present in cases that are in the very low or low IPSS‐R risk groups. Thus, monitoring CLEC12A expression on the CD34^+^CD38^−^ compartment longitudinally in a cohort of ICUS patients could add important knowledge to the diagnostic value of this easily applicable flow cytometry‐based assay.

Several recent studies have investigated the intraclonal heterogeneity in various myeloid malignancies. Using whole genome sequencing of myeloblasts from secondary AML (sAML) with subsequent deep resequencing of paired skin, MDS and sAML samples, Walter *et al* (2012) showed MDS and sAML to be highly oligoclonal with outgrowth of smaller MDS clones acquiring additional mutations at the time of progression to sAML. In addition, Lindsley *et al* (2015) recently examined 17 paired MDS/sAML samples and found AML‐related mutations in myeloid transcription factor and signalling genes down to an allele frequency of 1% at the time of MDS (Lindsley *et al*, 2015). Thus, studying even minute fractions of stem cells with aberrant expression of, for example, CLEC12A by flow cytometry could be of high relevance considering this pronounced intraclonal diversity in the myelodysplastic neoplasm. In this regard, future studies are warranted to evaluate the possible genetic/epigenetic differences between the CD34^+^CD38^−^CLEC12A^+/−^ subsets in MDS. One interesting approach would be studies to evaluate if CLEC12A positive CSCs are more prone to transformation to AML, e.g. through activated proliferative pathways and/or a different mutational status. Such studies might also shed light on a possible functional role of CLEC12A in myeloid neoplasms.

The most interesting hallmark of CLEC12A is its absence on the CD34^+^CD38^−^ cell subset from normal donors. Consequently, CLEC12A has been proposed as a potential target in the future treatment of AML because residual normal HSCs would be spared. Basic research is currently investigating different approaches in this regard: for example, Lu *et al* (2014) developed a novel immunotherapeutic bispecific antibody for the recruitment of cytotoxic T‐cells to CLEC12A‐positive AML cells. As mentioned, our results show that CLEC12A does not have the potential of distinguishing CSCs from their normal counterparts in MDS. Despite this, the CD34^+^CD38^−^CLEC12A^+^ cells might represent a more proliferative subclone in the pool of malignant cells. Hence speculatively, targeting CLEC12A in MDS would not eradicate all malignant clones, but could theoretically target a potentially more aggressive subclone.

## Authorship contributions

M.T.P., L.N. and A.S.R. designed the study, performed the experiments and analysed the data. M.T.P. wrote the manuscript with input and critical reviews from L.N., P.H., E.K. and A.S.R.. E.K. performed and analysed the FISH‐experiments. G.B.K. performed morphological examination of MDS BM samples. G.D.B. provided the anti‐CLEC12A hybridoma. All authors read and approved the final manuscript.

## Disclosure of conflicts of interest

The authors declare no competing interests.
